# Food Environment Around Schools in a Capital City in Southern Brazil: Changes in the Availability of Commercial Food Establishments Between 2012 and 2019

**DOI:** 10.3390/ijerph22060877

**Published:** 2025-05-31

**Authors:** Lidiamara Dornelles de Souza, Elizabeth Nappi Corrêa, Adalberto Aparecido dos Santos Lopes, Cristine Garcia Gabriel, Francisco de Assis Guedes de Vasconcelos

**Affiliations:** 1 Postgraduate Program in Nutrition, Federal University of Santa Catarina, Florianópolis 88040-370, SC, Brazil; f.vasconcelos@ufsc.br; 2 Center for Health Sciences, Department of Nutrition, Federal University of Santa Catarina, Florianópolis 88040-370, SC, Brazil; elizabeth.nappi@ufsc.br (E.N.C.); cristine.gabriel@ufsc.br (C.G.G.); 3 Urban Health Observatory, Federal University of Minas Gerais, Belo Horizonte 31270-901, MG, Brazil; adalberto.lopes@posgrad.ufsc.br

**Keywords:** built environment, community food environment, school environment, food retail, school health

## Abstract

Objective: The objective of this article was to perform a comparative analysis of the changes in the availability of commercial food establishments around the schools in a Brazilian capital city. Methods: This is a comparative analysis between two cross-sectional panels performed in 2012 and 2019 in Florianópolis, Santa Catarina, Brazil. Secondary data from different sources were used. The location data of schools and establishments were georeferenced. For the analysis of the surroundings of schools, a network buffer of 800 m was considered. The establishments were classified based on the foods they primarily sold: fresh, mixed and ultra-processed foods. The chi-square and Wilcoxon tests were applied for the analyses, considering a significance level of 95% (*p* < 0.05). Results: The number of food establishments around schools increased by 63%. There was a statistically significant growth in mixed (*p* = 0.008) and ultra-processed (*p* = 0.024) food- selling establishments. Conclusion: We conclude that the increase in establishments selling ultra-processed foods around schools at the expense of establishments selling fresh food deserves attention. This condition requires public policies that help promote healthier environments, thus contributing to the health of schoolchildren.

## 1. Introduction

The food environment, defined as the physical, economic, political and sociocultural environment in which individuals interact and are consequently influenced to choose, prepare and consume food [1], has been studied with the aim of understanding its relationship with, and its role as a determinant of, individuals’ food choices, acting as a facilitator or barrier, influencing the quality of food and consequently health levels [2,3,4]. Evidence in the literature indicates that multiple physical, economic, political and sociocultural aspects determine which foods are available, accessible and desirable, contributing to the development of individuals’ eating habits [5,6,7,8,9]. The physical aspects of the food environment include the community food environment, defined in a pioneering way by Glanz et al. [10] as being the number, type, location and accessibility of food establishments in a given community.

Some studies on the community food environment, considering the child and adolescent populations, have carried out assessments over the last ten years, focusing on the areas surrounding schools [11,12,13]. Scientific evidence shows that the school environment can influence the food choices of schoolchildren, since commercial food establishments (places that sell food products directly to the consumer), especially those that sell low-cost, high-energy-density foods, are usually located close to schools and, consequently, schoolchildren would be exposed to such products offered in those locations [11,12,13]. Furthermore, the environment around schools seems to differ from other food environments, considering that schoolchildren may not be directly influenced by their family during the journey from home to school [14,15].

Furthermore, scientific evidence also shows that the food environment around schools contributes in different ways to the development of children’s and adolescents’ body weight [16,17,18]. A systematic review of the literature, which included 31 observational studies published between 2009 and 2019, reviewed the association between the food environment around schools and obesity in children and adolescents [19]. A direct association between the proximity or density of establishments around schools and overweight/obese children and adolescents was found [19], showing that the presence of commercial food establishments in the neighborhood of schools can be a potentiating or protective factor for obesity, depending on schoolchildren’s access to those places, as well as the types of food offered there.

In this connection, studies carried out in developed countries, for example, Belgium, Canada, Spain, Netherlands and the United States of America, have shown that establishments selling foods considered unhealthy, with a predominance of ultra-processed foods [20], were more frequently present near schools located in areas with lower socioeconomic status than in areas with higher socioeconomic status [21,22,23]. However, studies carried out in Brazil evaluating the surroundings of public and private schools revealed a predominance of establishments that primarily sell ultra-processed foods, regardless of the neighborhood income and the location of the schools, which exposes children and adolescents to an unhealthy food environment and may contribute to the availability of inadequate food choices [24,25,26]. In Brazil, there is no regulation regarding the presence of food establishments around schools in the country.

In summary, the assessment of the food environment to which schoolchildren are exposed can be an important factor to help support public policies on this issue, thus contributing to health promotion [27,28,29]. Therefore, studies on this topic are necessary, especially in low- and middle-income countries such as Brazil, where studies on the food environment and schoolchildren are still scarce and concentrated in a few locations in the country. Hence, this article aims to perform a comparative analysis of the changes that have occurred in the community food environment in the neighborhood of public and private schools in a Brazilian capital between 2012 and 2019.

## 2. Materials and Methods

This is a comparative analysis study of two cross-sectional panels, carried out in public and private elementary schools in Florianópolis, the capital of the state of Santa Catarina. The municipality had an estimated population of 576,361 inhabitants in 2024, a demographic density of 796.05 inhabitants/km^2^ and 0.847 MHDI (Municipal Human Development Index). In 2010, the estimated population was 421,340 inhabitants and the population density was 623.69 inhabitants/km^2^ [30].

This investigation is linked to the “Study of Prevalence of Obesity in Children and Adolescents in Florianópolis, SC (EPOCA)”, which is characterized as research of repeated cross-sectional panels, carried out since 2002 [31,32,33,34]. For this study, only data from the 2012 and 2019 panels were used, given that information regarding the food environment was incorporated from the 2012 panel.

As for the selection of schools, in both panels, they were initially divided into 10 strata, according to the administrative regions of the city of Florianópolis and the type of school (public or private). In each stratum, the sampling units were selected randomly, totaling 30 schools in each panel. In the 2012 panel, out of the 30 schools participating in the survey, 11 were private, 9 were municipal, 9 were state schools and one was a federal school [31,32]; in the 2019 panel, 11 were private, 8 were municipal and 11 were state schools [33,34]. In our study, the schools that were included in both panels were considered, totaling 11 schools: 3 private, 3 municipal and 5 state schools.

To capture information about the existence and location of commercial (formal) establishments selling ready-to-eat food and meals, a combination of sources was used to ensure coverage of the largest number of establishments. The information was obtained through secondary data from government databases (municipal and state) and private institutions (online telephone directories) using the National Classification of Economic Activities (CNAE) [35].

At the end of the confirmation and/or completion process, the commercial food establishments found were initially grouped into 15 categories: butcher, fishmonger, restaurant, snack bar, candy and sweets retailer, bakery, hypermarket, supermarket, market/grocery store, fruit and vegetable retailer, street market, food retailer, dairy retailer, convenience store and street vendor [35].

Subsequently, the commercial food establishments were regrouped and classified into three categories: (i) establishments that sell fresh foods; (ii) establishments that sell ultra-processed foods; and (iii) establishments that sell mixed foods. To determine this classification, pre-established criteria were used that delineate establishments where selling fresh or minimally processed foods represents more than 50% of their sales—in other words, selling, predominantly, products considered healthier than more processed alternatives—from establishments where sales of ultra-processed foods represent more than 50% of total food sales (a predominance of products considered unhealthy). This classification was determined according to the criteria defined by the Interministerial Chamber of Food and Nutrition Security (CAISAN) for the state of Santa Catarina [36], which is based on the categories present in the Food Guide for the Brazilian Population [37]. Following the regrouping process, the establishments were categorized as shown in Table 1.

To analyze the food environment around schools, the methodological procedure of constructing an 800-m network buffer around each of the 11 schools investigated was used [19]. The location of the school was used as the centroid point. To spatialize the data (create a spatial reference based on geographic coordinates), the full addresses of the schools and of the food establishments were entered into the Google Earth^®^ software. By creating the buffer zones, it was possible to count and spatially visualize each type of food establishment around the schools and the changes that occurred between 2012 and 2019.

Data are presented in absolute (n) and relative (%) frequency or mean (M) and standard deviation (SD). The variation in the proportion of types of establishments during the period from 2012 to 2019 was calculated by decreasing the percentage value corresponding to that type of establishment in 2019 by the equivalent percentage value in 2012 (∆% = %2019 − %2012). The proportional variation of establishments during the same period was calculated using the following equation: ∆% = ((n2019 − n2012)/n2012) × 100.

To compare the distribution of establishments selling predominantly fresh, mixed and ultra-processed products in 2012 and 2019, the chi-square test was used. The Wilcoxon test was used to evaluate the pairwise comparison of the number of commercial food establishments around 11 schools in Florianópolis (SC), according to the type of establishment and the predominance of foods sold, in 2012 and 2019. The significance level adopted was 95% (*p* < 0.05). The analyses were performed using Microsoft Excel v. 2021 (Microsoft Corporation, Redmond, WA, USA) and IBM SPSS for Statistics v.26 software (IBM, Armonk, NY, USA).

For the spatial descriptive analysis (spatial visualization of the absolute availability of commercial food establishments around the schools), maps were created using ArcGis^®^ Desktop 10.5 software (Esri, Redlands, CA, USA).

## 3. Results

Data were collected from 7834 commercial food establishments in the municipality, 2693 in 2012 and 5141 in 2019 (∆% = 91%). The most prevalent establishments in both periods were restaurants (2012: 41.0%; 2019: 30%) and snack bars (2012: 27.2%; 2019: 27.6%). Establishments defined as convenience stores (−1.5%), bakeries (3.5%), restaurants (11.0%), candy retailers (−0.2%) and street markets (−0.5%) showed a negative variation in the proportion of types of establishments during the period. The proportion of hypermarkets did not vary between 2012 and 2019. The other establishments showed an increase in the proportion of establishments in 2019 compared to 2012. Retail establishments of food products stand out, representing 1.4% (n = 37) of the establishments evaluated in 2012, and representing 11.7% (n = 604) of the establishments in 2019 (Table 2).

In the analysis of the proportional variation in the number of establishments during the period, a 51% reduction in the number of convenience establishments was observed (n = 55 in 2012; n = 27 in 2019). All other establishments showed a significant increase in the number of stores between 2012 and 2019. During that period, the increase in the number of food retail establishments (∆% = 1532%), dairy retailers (∆% = 413%), street vendors (∆% = 312%) and fruit and vegetable vendors (∆% = 282%) stands out (Table 2).

Table 3 shows the distribution of commercial food establishments in Florianópolis (SC), according to the predominance of foods sold in 2012 and 2019. A statistically significant difference was observed in the proportion of types of establishments during the period (z = 6.192; *p* = 0.045). An increase in the proportion of fresh food and ultra-processed establishments (1.2% each) and a reduction in the proportion of mixed establishments (−2.3%) were observed. However, when considering the proportional variation in the number of establishments during the period, there was an increase of 133% in establishments with a predominance of fresh foods, 98% in ultra-processed establishments and 84% in mixed establishments (Table 3).

The comparison of the number of commercial food establishments in the neighborhood of schools in Florianópolis is presented in Table 4. The vicinity of 11 schools (n = 8 public and n = 3 private) was evaluated during the years 2012 and 2019. The total number of establishments in the vicinity of schools increased during the period (*p* = 0.003); there were 225 establishments in 2012 (M = 20.5; SD = 22.7) and 380 in 2019 (M = 34.5; SD = 27.9). Significant increases were observed in the number of street vendors (*p* = 0.016), snack bars (*p* = 0.016), mini-markets (*p* = 0.007), restaurants (*p* = 0.028) and food retailers (*p* = 0.005). The number of bakeries decreased during this period, reaching zero in 2019 (*p* = 0.011). When analyzing the establishments according to the predominance of foods sold, there was a statistically significant increase in the number of mixed establishments (*p* = 0.005) and in the predominant sale of ultra-processed foods (*p* = 0.014).

**Figure 1:**
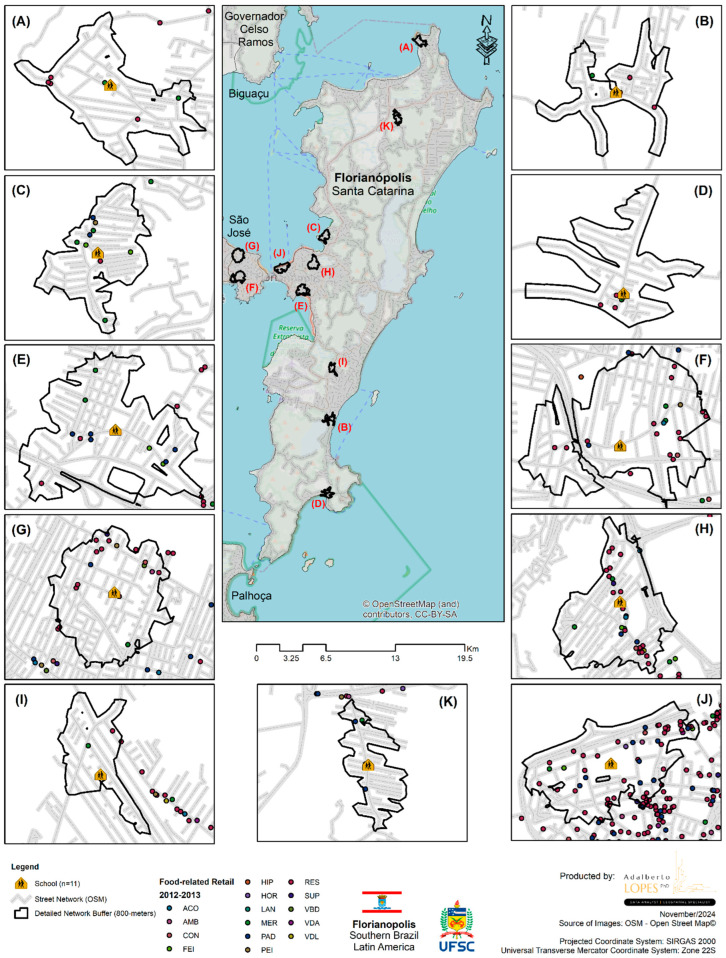
Spatial distribution by type of commercial food establishment around 11 schools in the city of Florianópolis (SC) in 2012.

**Figure 2:**
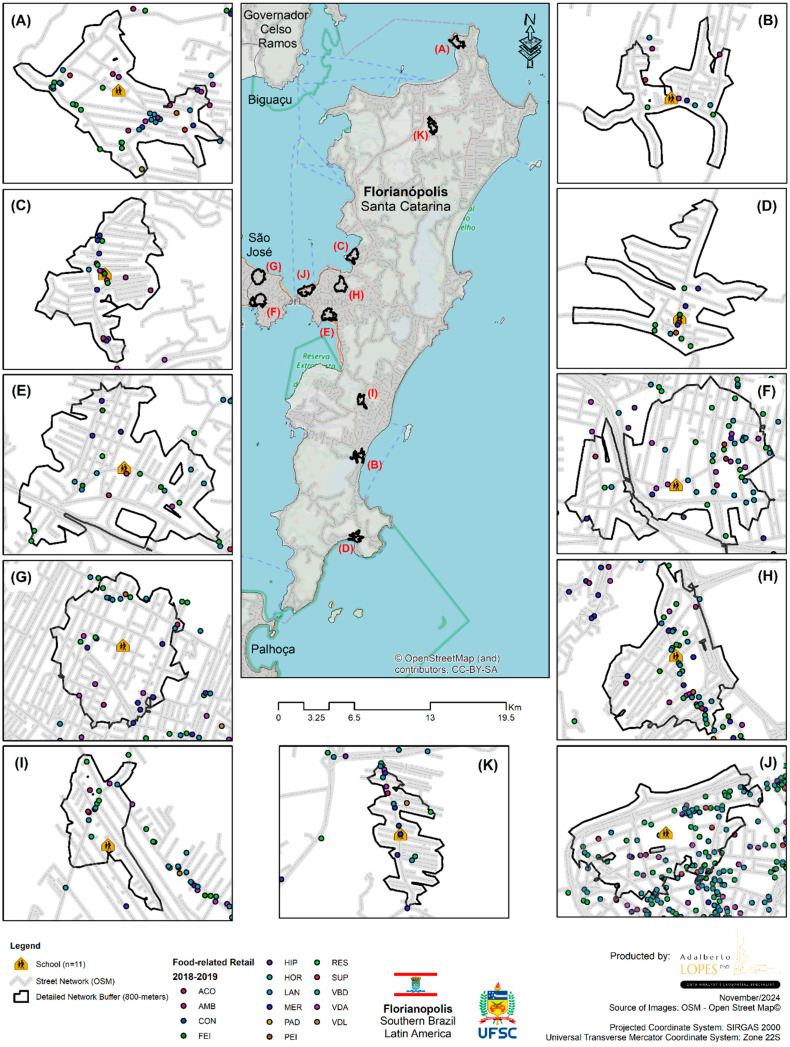
Spatial distribution by type of food establishment in the neighborhood of 11 schools in the city of Florianópolis (SC) in 2019.

Figure 1 and Figure 2 illustrate the spatial distribution by types of food establishments in the neighborhood of 11 schools in 2012 and 2019, respectively.

**Figure 3:**
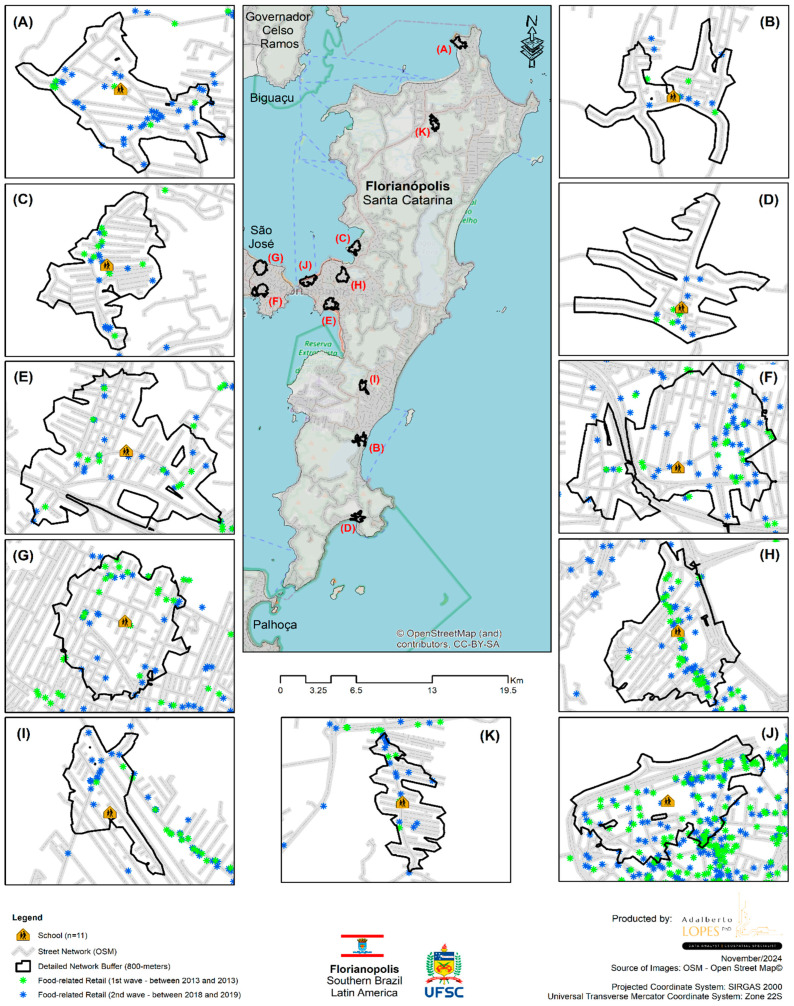
Spatial distribution of food establishments around 11 schools in the city of Florianópolis (SC) in 2012 and 2019.

The changes that occurred between 2012 and 2019 in the number of food establishments around the 11 schools are shown in Figure 3. It is possible to see that the biggest changes occurred around the schools labelled A, J and K.

## 4. Discussion

In the present study, it was observed that, between 2012 and 2019, in the areas surrounding the 11 schools evaluated in 2012 and 2019, there was a significant increase in the number of street vendors and snack bars, reflecting the increase in establishments that predominantly sell ultra-processed foods. On the other hand, establishments that predominantly sell fresh foods showed a slight increase, but there were no retailers selling fresh produce, for example, in the area studied.

The results of this study showed that in the city of Florianópolis (SC) there was an increase in the number of establishments that sell natural foods proportional to the number of establishments selling ultra-processed foods. A similar finding was also reported in a study carried out in the city of Melbourne (Australia) that evaluated the variation in the density of food establishments from 2008 to 2016 [38]. The authors found that the number of food establishments increased driven by a growing density of establishments considered “unhealthy” and “healthy” [38].

Unlike the present study, authors who evaluated changes in the food trade, considering the predominant type of food, found an increase in establishments selling ultra-processed foods to the detriment of other types of establishments [39,40,41]. A study that evaluated the characteristics of food establishments between 1990 and 2014 in the United States demonstrated that, throughout the period assessed, the density of establishments that primarily sold ultra-processed foods was always higher than that of those that sold foods considered healthy [41].

In Brazil, a study that assessed changes in the availability of food establishments in a capital city over a ten-year period showed a 154% growth in “unhealthy” establishments compared to a 32% increase in establishments considered healthy; the authors thus considered a worsening of the community food environment over the period assessed [40]. In New York, United States of America, a study that reviewed the evolution of the availability of food establishments considered unhealthy over a twenty-year period concluded that the average number of unhealthy food outlets doubled, going from approximately three establishments of this type per census tract in 1990 to approximately six in 2010 [39].

Several studies indicate that, in general, the availability of commercial establishments classified as selling fresh food and mixed food contributes to the consumption of healthy foods by the population [42,43,44]. In contrast, ultra-processed establishments contribute to the increased consumption of foods with low nutritional quality, which can lead to increased rates of obesity and other chronic non-communicable diseases [45,46,47,48].

In this study, due to the significant increase in the number of street vendors and snack bars (110%) and in the number of mini-markets, restaurants and retailers mainly selling food (77%), a significant increase in ultra-processed and mixed commercial establishments was observed in the vicinity of schools. The growth in the number of fresh food establishments was only 33%. In relation to this environment, the main hypothesis of the studies on this topic is that the presence of food establishments in this area, depending on levels of access and the characteristics of the food sold in a particular location, can be a facilitating or protective factor for overweight/obese schoolchildren [19,49,50,51,52,53,54].

National and international studies have demonstrated the predominance of commercial establishments that sell primarily ultra-processed foods in the neighborhood of public or private schools [12,24,25,26,55,56,57]. These findings ultimately highlight the exposure of children and adolescents to a food environment that is favorable to the consumption of foods considered unhealthy, most of which are high in caloric density and low in nutrients. It is noteworthy that, among the multiple and complex determinants that influence healthy school-aged children’s diets, in addition to those investigated and already discussed in this study, other factors such as educational level, socioeconomic status and parents’ food and nutritional literacy and knowledge rates have been highlighted in the literature. Hoteit et al. (2023), for example, reported that parents with high food and nutritional literacy were more likely to provide healthier food choices among adolescents in 10 Arab countries [58].

In Madrid (Spain), a study evaluated the surroundings of 1321 schools and associated them with the socioeconomic level of the neighborhood where they were located [23]. A total of 95% of the schools had unhealthy food establishments operating in their surroundings. Furthermore, schools located in areas of low socioeconomic level had a 62% higher number of establishments considered to be selling unhealthy food, when compared to schools located in areas of medium socioeconomic level [23]. Also in Spain, in Barcelona, Londoño-Cañola et al. (2023) examined the surroundings of 22 schools with the aim of evaluating the availability of food establishments in the surroundings of schools and the association with the socioeconomic level of the neighborhood where they were located [55]. The authors found that 95% of the establishments located in a 400 m buffer zone, with the school being the centroid point, sold primarily unhealthy foods. They also found a positive association between schools located in neighborhoods with higher socioeconomic status and greater availability and accessibility of healthy foods [55].

A few studies conducted in Brazil aimed to evaluate the availability of food establishments in the neighborhood of schools, in the framework of the administrative condition of the school (public or private) and the socioeconomic level of their location residents [12,56]. In a study conducted in the city of Niterói, Rio de Janeiro, the authors analyzed and compared the types of food sold in the neighborhood of 30 private and 26 public elementary schools [12]. The amount of ultra-processed foods sold in the vicinity of the schools was statistically higher than for other food categories (fresh and processed food), with no statistically significant difference in the categories of food sold between public and private schools [12]. In a study conducted in the city of Recife, Pernambuco, the authors described the community food environment near schools and its association with the socio-environmental vulnerability of the territory in which they were located [56]. The grouping pattern of establishments that included street vendors, snack bars and kiosks was described as predominant around schools and representative of the sale of foods considered unhealthy in the environment assessed [56]. In our study, due to the small number of private schools included at both the periods of time investigated, it was decided to disregard the analysis by the administrative type of school.

An important point in this study was that during the period assessed there was a 133% increase in the proportional variation of the number of establishments classified as selling fresh food in the city of Florianópolis. However, when examining the surroundings of the schools included in the study, this type of establishment showed a decrease in its proportional variation, evidencing an inequality in the availability of healthy foods. In this connection, the presence of food deserts and food swamps has been discussed within the school environment. Food deserts are defined as areas characterized by low availability and consequently limited access to foods considered healthy, such as fruits, vegetables and greens [59]. Food swamps are defined as areas that have a high density of establishments that primarily sell ultra-processed foods [60].

The presence of food deserts and swamps in the school environment appears to be a factor influencing the food choices of children and adolescents. Studies show that the higher density of commercial establishments in the school environment that primarily sell unhealthy foods is associated with nutritionally deficient food choices, which may contribute to inadequate food consumption and consequent nutritional disorders in that population [19,61]. A literature review study analyzed the association between food in the school environment and the purchase of unhealthy foods among adolescents aged 10 to 19 years. The authors found evidence of a positive association between school environments that had a high density of commercial establishments that primarily sold unhealthy foods or the presence of this type of establishment on the route to/from school and a higher frequency of unhealthy foods purchases among adolescents [61].

In Brazil, school meals in the public education system are governed by a legislative framework that aims to promote healthy eating habits [28,62,63]. In addition, several states and municipalities already offer legal provisions to ensure the supply of healthy foods in public and private school cafeterias [27,64]. Therefore, the availability of food within schools, which comprises part of the so-called organizational food environment [65], has received legislative support in recent years aimed at improving, in terms of nutrition and habit formation, what is offered and made available to students, requiring a closer look at the school environment. In this context, coordination between civil society and public authorities is essential to enhance strategies that aim to promote healthier food environments in order to consolidate food assurance and nutritional security for the population.

One of the strengths of this work was the comparative analysis of the availability of establishments, both from the general point of view of the municipality and, mainly, of the surroundings of the participating schools. Another positive point was the use of the network buffer that considers the actual possibilities of movement through the environment evaluated.

As a limitation, we highlight the use of different sources of secondary data to locate food establishments in the two panels, a fact that may generate inaccuracies. In an attempt to mitigate this limitation, in 2012, different strategies were used to complement/check the information, such as consulting printed and online telephone directories available in the municipality and consulting the websites of official organizations. In 2019, a virtual check of the information was carried out, giving greater reliability to the data extraction. However, further studies are required to carry out on-site audits to check these establishments. In addition, it is important to highlight the issue related to the mapping of street markets, mainly because their frequency can change depending on the location, further affecting the availability of fresh food. Another limiting factor was the small number of schools included in both panels, which restricted the sample to 11 schools.

## 5. Conclusions

The outcome of this study presents an overview of the changes that occurred in the availability of food establishments between 2012 and 2019 in the city of Florianópolis, SC. It also presents the visualization of these changes in the food environment around public and private schools, allowing for a reflection on students’ access to different types of food.

We concluded that the increase in the proportion of establishments that sell natural foods in the municipality as a whole was not a reality in the surroundings of the schools evaluated. In this specific environment, there was an increase in the number of outlets selling ultra-processed and mixed food. We hope that the results found in the study can help guide effective public policies for schoolchildren aimed at health promotion and the prevention of obesity.

## Figures and Tables

**Table 1:** Categories of commercial food establishments according to the criteria defined by CAISAN.

Establishments Fresh Food	Establishments Ultra-Processed	Establishments Mixed
Butcher’s shop Street market Fishmonger’s shop Fresh produce retailer	Snack bars Convenience store Candy retailer Street vendor	Hypermarket Market/grocery store Bakery Restaurant Supermarket Food retailer Dairy retailer

**Table 2:** Distribution of food establishments in Florianópolis (SC), according to the National Classification of Economic Activities (CNAE) and the assessment years 2012 and 2019.

	2012		2019		Variation in the Proportion of Types of Establishments During the Period	Proportional Variation in the Number of Establishments During the Period
	n	%	n	%	∆% = %2019 − %2012	∆% = (n2019 − n2012/n2012) × 100
Butcher	27	1.0	73	1.4	0.4	170%
Street vendor	59	2.2	243	4.7	2.5	312%
Convenience store	55	2.0	27	0.5	−1.5	−51%
Hypermarket	5	0.2	12	0.2	0.0	140%
Snack bar	733	27.2	1418	27.6	0.4	93%
Minimarket	280	10.4	555	10.8	0.4	98%
Bakery	198	7.4	202	3.9	−3.5	2%
Restaurant	1103	41.0	1543	30.0	−11.0	40%
Supermarket	30	1.1	95	1.8	0.7	217%
Candy retailer	47	1.7	79	1.5	−0.2	68%
Street markets	48	1.8	66	1.3	−0.5	38%
Fruit and vegetable retailer	28	1.0	107	2.1	1.1	282%
Fishmonger	35	1.3	76	1.5	0.2	117%
Dairy retailer	8	0.3	41	0.8	0.5	413%
Food retailer	37	1.4	604	11.7	10.3	1532%
Total number of establishments	2693	100.0	5141	100.0		91%

Legend: Orange-colored cells represent a positive proportional change and blue-colored cells represent a negative proportional change.

**Table 3:** Distribution of food establishments in Florianópolis (SC), according to the predominance of foods sold, 2012 and 2019.

	2012		2019		Variation in the Proportion of Types of Establishments During the Period	Proportional Variation in the Number of Establishments During the Period
	n	%	n	%	∆% = %2019 − %2012	∆% = (n2019 − n2012/n2012) × 100
Fresh Food	138	5.1	322	6.3	1.2	133%
Mixed	1661	61.7	3052	59.4	−2.3	84%
Ultra-processed	894	33.2	1767	34.4	1.2	98%
Total number of establishments	2693	100.0	5141	100.0		

Legend: Orange-colored cells represent a positive proportional change and blue-colored cells represent a negative proportional change.

**Table 4:** Comparison of food establishments around 11 schools in Florianópolis (SC), according to the type of establishment and the predominance of food sold, 2012 and 2019.

	2012		2019		Wilcoxon (Z-Test)	*p*
	Mean ± SD	Total	Mean ± SD	Total		
Butcher’s shop	0.4 ± 0.9	4	0.5 ± 0.7	6	−0.707	0.480
Street markets	0.6 ± 0.9	7	0.7 ± 1.0	8	−0.577	0.564
Fishmonger’s shop	0.3 ± 0.5	3	0.6 ± 0.7	7	−1.414	0.157
Fruit and vegetable retailer	0.1 ± 0.3	1	0.0 ± 0.0	0	−1.000	0.317
Fresh Establishments	1.4 ± 1.5	15	1.9 ± 1.4	21	−1.294	0.196
Snack Bar	5.3 ± 6.7	58	10.5 ± 11.7	115	−2.405	**0.016**
Convenience Store	0.4 ± 0.5	4	0.1 ± 0.3	1	−1.342	0.180
Candy Retailer	0.1 ± 0.3	1	0.3 ± 0.6	3	−0.816	0.414
Street Vendors	0.2 ± 0.4	2	1.5 ± 1.5	16	−2.410	**0.016**
Ultra-processed food establishments	5.9 ± 7.2	65	12.3 ± 12.3	135	−2.451	**0.014**
Hypermarket	0.0 ± 0.0	0	0.2 ± 0.4	2	−1.414	0.157
Minimarket/grocery store	1.9 ± 1.0	21	4.0 ± 2.1	44	−2.699	**0.007**
Bakery	2.4 ± 2.9	26	0.0 ± 0.0	0	−2.536	**0.011**
Restaurant	8.5 ± 11.5	93	10.6 ± 12.1	117	−2.203	**0.028**
Supermarket	0.3 ± 0.5	3	0.8 ± 1.3	9	−1.186	0.236
Dairy retailer	0.1 ± 0.3	1	0.4 ± 0.7	4	−1.134	0.257
Food retailer	0.1 ± 0.3	1	4.4 ± 3.2	48	−2.812	**0.005**
Mixed Establishments	13.2 ± 14.7	145	20.4 ± 15.4	224	−2.809	**0.005**
Total number of establishments	20.5 ± 22.7	225	34.5 ± 27.9	380	−2.937	**0.003**

## Data Availability

The original contributions presented in this study are included in the article. Further inquiries can be directed to the corresponding authors.
